# Expression of concern: Emergent periodicity in the collective synchronous flashing of fireflies

**DOI:** 10.7554/eLife.101848

**Published:** 2024-10-03

**Authors:** Timothy E Behrens, Detlef Weigel

Raphael Sarfati, Kunaal Joshi, Owen Martin, Julie C Hayes, Srividya Iyer-Biswas, Orit Peleg. 2023. Emergent periodicity in the collective synchronous flashing of fireflies. *eLife* 12:e78908. doi: https://doi.org/10.7554/eLife.78908

Published 13 March 2023

This editorial expression of concern is to alert readers to issues relating to the experimental data included in this publication.

The editors are willing to re-review an updated version but so far the authors have not reached agreement on a potential Correction notice or an updated version of the submission.

We will issue an update in due course.

