# Correction: Emergent periodicity in the collective synchronous flashing of fireflies

**DOI:** 10.7554/eLife.109449

**Published:** 2025-10-15

**Authors:** Raphael Sarfati, Kunaal Joshi, Owen Martin, Julie C Hayes, Srividya Iyer-Biswas, Orit Peleg

**Keywords:** Other

Sarfati R, Joshi K, Martin O, Hayes JC, Iyer-Biswas S, Peleg O. 2023. Emergent periodicity in the collective synchronous flashing of fireflies. *eLife* **12**:e78908. doi: 10.7554/eLife.78908.

Published 13 March 2023

This correction supersedes the information provided in the Expression of Concern related to this article (Sarfati et al., 2023; Behrens and Weigel, 2024).

This correction addresses issues that were identified during preparation of the Version of Record. These corrections have been applied to the author accepted manuscript, and a proofed Version of Record based on the corrected article will follow in due course.

## Dataset corrections

After the acceptance of the paper, we discovered that one of the firefly flash recordings had been accidentally mislabeled. The problematic dataset in question involved an experiment with 20 fireflies. On the evening of June 7, 2020, members of the Peleg lab conducted two separate tent experiments simultaneously, one of which involved a set of LEDs. This setup was not part of the experiment reported in the paper. The LEDs were programmed to mimic the interburst intervals (Tb) observed in wild fireflies, incrementally adjusting the Tb parameter value to determine an optimal value for entrainment. This triggered us to carefully review our data, including the raw movies, the flash-time series, interburst interval (Tb) values, and our field notes, and we found the following corrections are required.

1. *Trimming the LED segment within 06072020* u. In the 20-firefly trial 06072020 u, which lasted about 69 minutes in the original dataset, the LEDs were switched on after about 30 minutes. We trimmed this recording to 15 minutes so that the LED segment is completely removed. We initially considered substituting the second 20-firefly recording from that night (06072020 c), but we retained 06072020 u (trimmed to 15 minutes) because it is needed for the protocol-mismatch correction described next in *Protocol Variation in Trials*.
2. *Protocol Variation in Trials*. The 20-firefly trial 06102021 c used a non-standard staging sequence (1–2-3-4-15-20 fireflies, instead of 1-5-10-15-20 fireflies, five minutes per stage) and is, therefore, not comparable to the other 20-firefly recordings. To keep a full set of three standard 20-firefly trials, we replaced 06102021 c with 06072020 c.
3. *Missing Single Firefly Trials*. Three out of the ten single-firefly recordings from 2021 had also been omitted from the original dataset, and they are now included.

We include below Figure 1, which provides a visual comparison of the original and corrected datasets, as well as a table summarizing the corrected dataset (Table 1) and the original dataset (Table 2). We also include both the original and corrected datasets themselves, for transparency.

**Figure 1.**
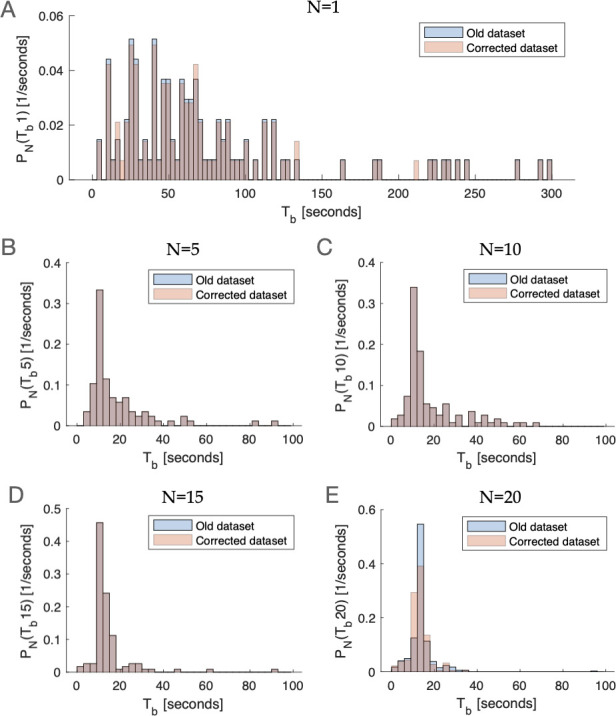
The probability distribution function of Tb (interburst interval) for experiments with 1 firefly (1ff) (**A**), 5ff (**B**), 10ff (**C**), 15ff (**D**), and 20ff (**E**), before and after making corrections to the dataset. Please note that the distributions of 5, 10, and 15ff remain unchanged. The bin size is set to 3 seconds.

**Table 1.** A table summarizing the corrected dataset.

#ff	Date label	Relevant time period	Duration in minutes
1	06012020u	260s - 4353s	68
1	06022020u	688s - 5520s	81
1	06032020a_1	152s - 4186s	67
1	06032020a_2	100s - 1833s	29
1	06042020a_1	102s - 3858s	63
1	06042020a_2	86s - 1852s	29
1	06112020c	160s - 5271s	85
1	06072021u^*^	3s - 1681s	28
1	06102021c^*^	10s - 300s	5
1	06122021c^*^	10s - 300s	5
5	06042020u	2111s - 2759s	11
5	06072020u	824s - 1410s	10
5	06082020u	939s - 1550s	10
10	06042020u	2799s - 3711s	15
10	06072020u	1450s - 2013s	9
10	06082020u	1590s - 2134s	9
15	06042020u	3751s - 4329s	10
15	06072020u	2053s - 2628s	10
15	06082020u	2174s - 2715s	9
20	06042020u	4369s - 5005s	11
20	06072020u^†^	2668s - 3558s	15
20	06072020c^*^	1922s - 2823s	15

* Data *added* to the dataset.

† Data *trimmed* in the updated dataset.

**Table 2.** A table summarizing the original dataset.

#ff	Date label	Relevant time period	Duration in minutes
1	06012020u	260s - 4353s	68
1	06022020u	688s - 5520s	81
1	06032020a_1	152s - 4186s	67
1	06032020a_2	100s - 1833s	29
1	06042020a_1	102s - 3858s	63
1	06042020a_2	86s - 1852s	29
1	06112020c	160s - 5271s	85
5	06042020u	2111s - 2759s	11
5	06072020u	824s - 1410s	10
5	06082020u	939s - 1550s	10
10	06042020u	2799s - 3711s	15
10	06072020u	1450s - 2013s	9
10	06082020u	1590s - 2134s	9
15	06042020u	3751s - 4329s	10
15	06072020u	2053s - 2628s	10
15	06082020u	2174s - 2715s	9
20	06042020u	4369s - 5005s	11
20	06072020u^†^	2668s - 6823s	69
20	06102021c*	1800s - 2099s	5

* Data *removed* from the dataset.

† Data *trimmed* in the updated dataset.

## Theoretical model validation

As a first sanity check, we have compared the original theory outputs to the original dataset minus the problematic data recordings (i.e., trimming 06072020 u to 15 minutes, and removing 06102021 c). As expected, the comparison between theory and experimental data is still robust (Figure 2).

**Figure 2.**
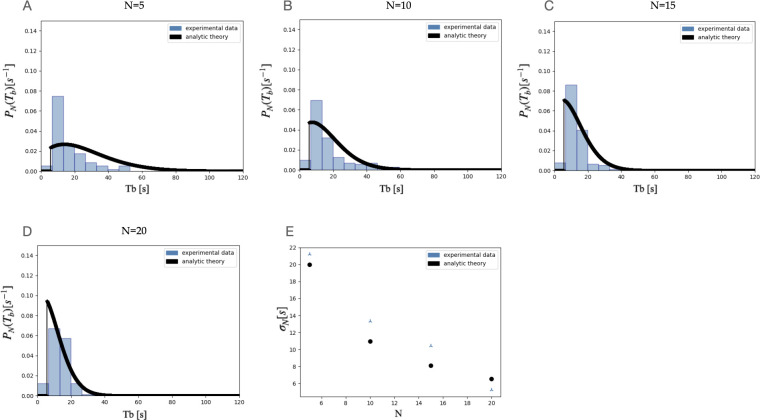
Comparison between theory and data for experiments, of the probability distribution function of the interburst interval (Tb), with 5ff (**A**), 10ff (**B**), 15ff (**C**), 20ff (D), and the resulting standard deviation of the interburst interval (Tb) (**E**), after removing the affected data points from the original dataset. The removal has no effect on the main conclusions.

Next, based on the revised single-firefly interburst intervals, we regenerated the input envelope used in the analytical theory. We have updated Figure 3 with the new theoretical distributions generated from this revised input envelope (Figure shown below).

## Computational model correction

We discovered that Figure 7 erroneously displayed the difference in medians between distributions instead of the intended two-sided Kolmogorov–Smirnov (K-S) test statistic.

During our review, we also found a minor error in the **Methods section, Agent-based simulation, Simulation parameters** and in the original caption of Figure 7, which stated that ten simulation trials were conducted. In fact, thirty trials were run in all cases, both in the published and updated figures.

## Updates to text and figures

**Methods section, Experimental data:**

Corrected text:

We observed 10 individual fireflies alone in the tent, over durations between 5 min and 85 min. We observed that although these fireflies produced flash trains at a frequency of about 2 Hz, the delay between successive trains was apparently randomly distributed, from a few seconds to tens of minutes. Then, we carried out three sets of experiments with 5, 10, 15, and 20 fireflies, using the segments between 9 minutes and 15 minutes. As previously reported, collective burst flashing only appears at about 15 fireflies.

Original text:

We observed 10 individual fireflies alone in the tent, over durations between 30 min and 90 min. We observed that although these fireflies produced flash trains at a frequency of about 2 Hz, the delay between successive trains was apparently randomly distributed, from a few seconds to tens of minutes. Then, we carried out 3 sets of experiments where the number of fireflies was increased to 5, then 10, then 15, then 20, each condition being maintained between 15 min and 30 min. As previously reported, collective burst flashing only appears at about 15 fireflies.

**Methods section, Experimental data correction:**

Added text:

After the paper’s acceptance, a small subset of data points was updated for the reasons described in the Supplementary Appendix. We repeated all analyses and confirmed that the findings are unaffected. Both the original and corrected datasets are publicly available.

Original text: n/a

**Methods section, Agent-based simulation, Simulation parameters:**

Corrected text:

For each set of parameters, we ran simulations for thirty trials of 200,000 timesteps each.

Original text:

For each set of parameters, we ran simulations for ten trials of 200,000 timesteps each.

**Discussion and concluding remarks section:**

Corrected text:

As shown in Fig. 3, the chosen values for beta, the additional fitting parameter introduced in the agent-based simulation, are: *β*=0.16, 0.16, 0.20 and 0.30 respectively for N=5, 10, 15, 20.

Original text:

As shown in Fig. 3, the chosen values for beta, the additional fitting parameter introduced in the agent-based simulation, are: *β*=0.18, 0.13, 0.12 and 0.64 respectively for N=5, 10, 15, 20.

**Figure 7 Caption:**

Corrected text:

The best values for each N=5,10,15,20 are *β*=0.16, *β*=0.16, *β*=0.20, *β*=0.30.

Original text:

The best values for each N=5,10,15,20 are *β*=0.18, *β*=0.13, *β*=0.12, *β*=0.64.

The corrected Figure 1 is shown here (only panel D was updated):

**Figure:**
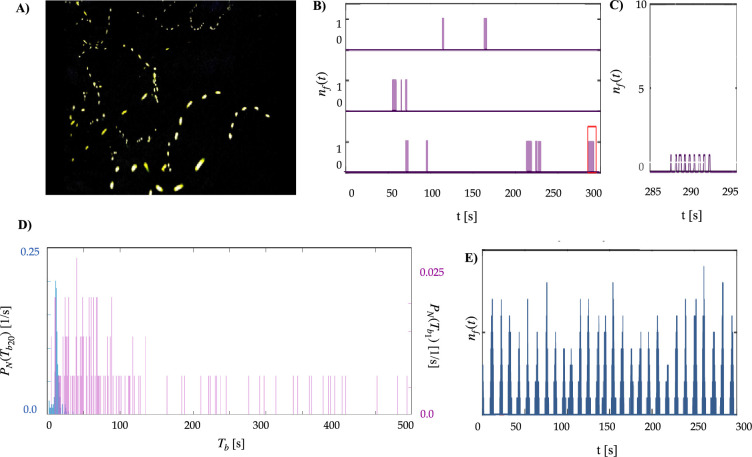


The originally published Figure 1 is shown for reference:

**Figure:**
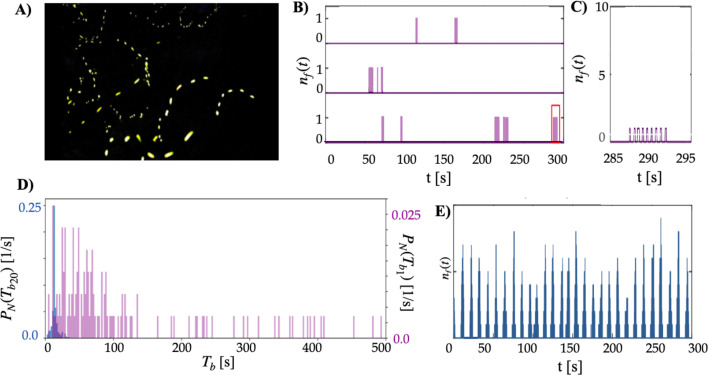


The corrected Figure 3 is shown here:

**Figure:**
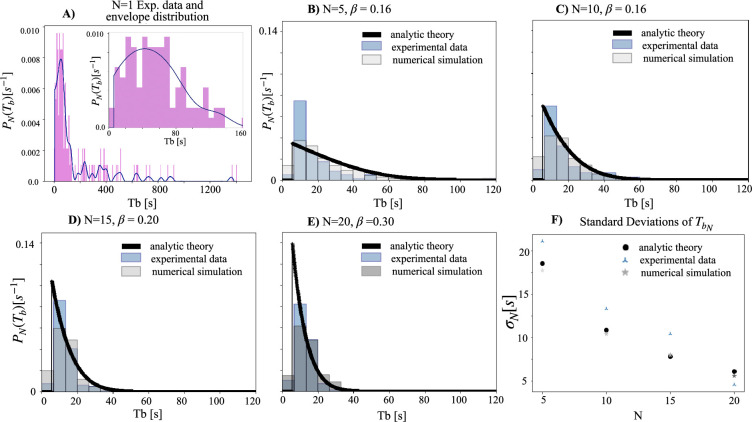


The originally published Figure 3 is shown for reference:

**Figure:**
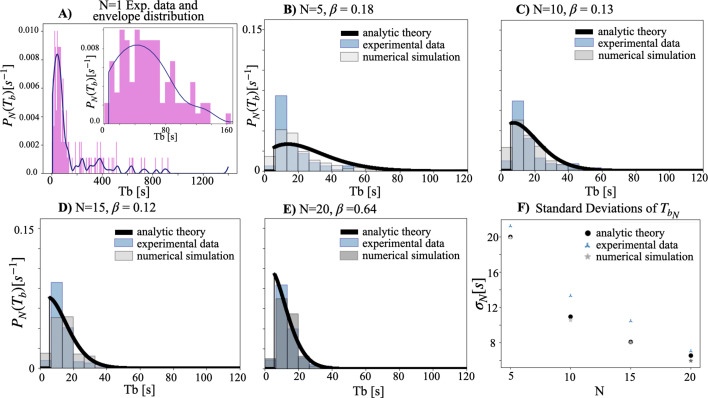


The corrected Figure 5 is shown here:

**Figure:**
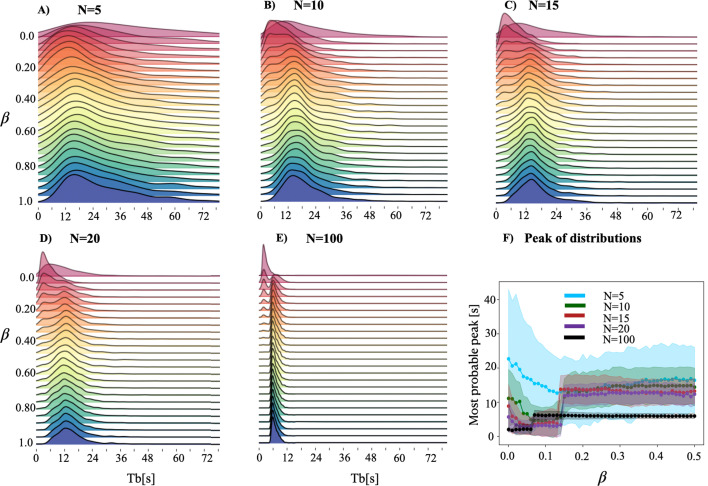


The originally published Figure 5 is shown for reference:

**Figure:**
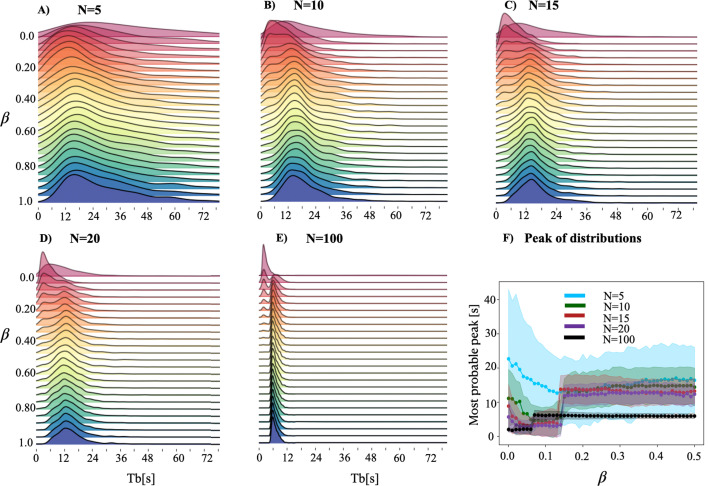


The corrected Figure 7 is shown here:

**Figure:**
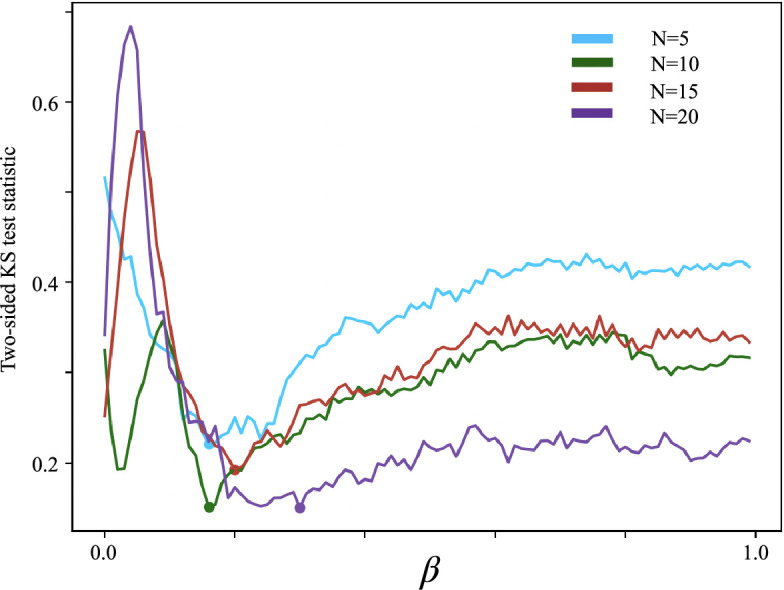


The originally published Figure 7 is shown for reference:

**Figure:**
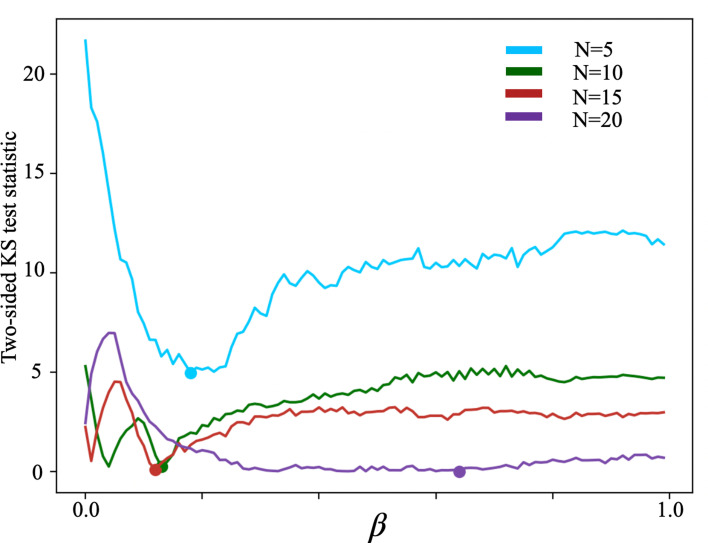


The article has been corrected accordingly.
